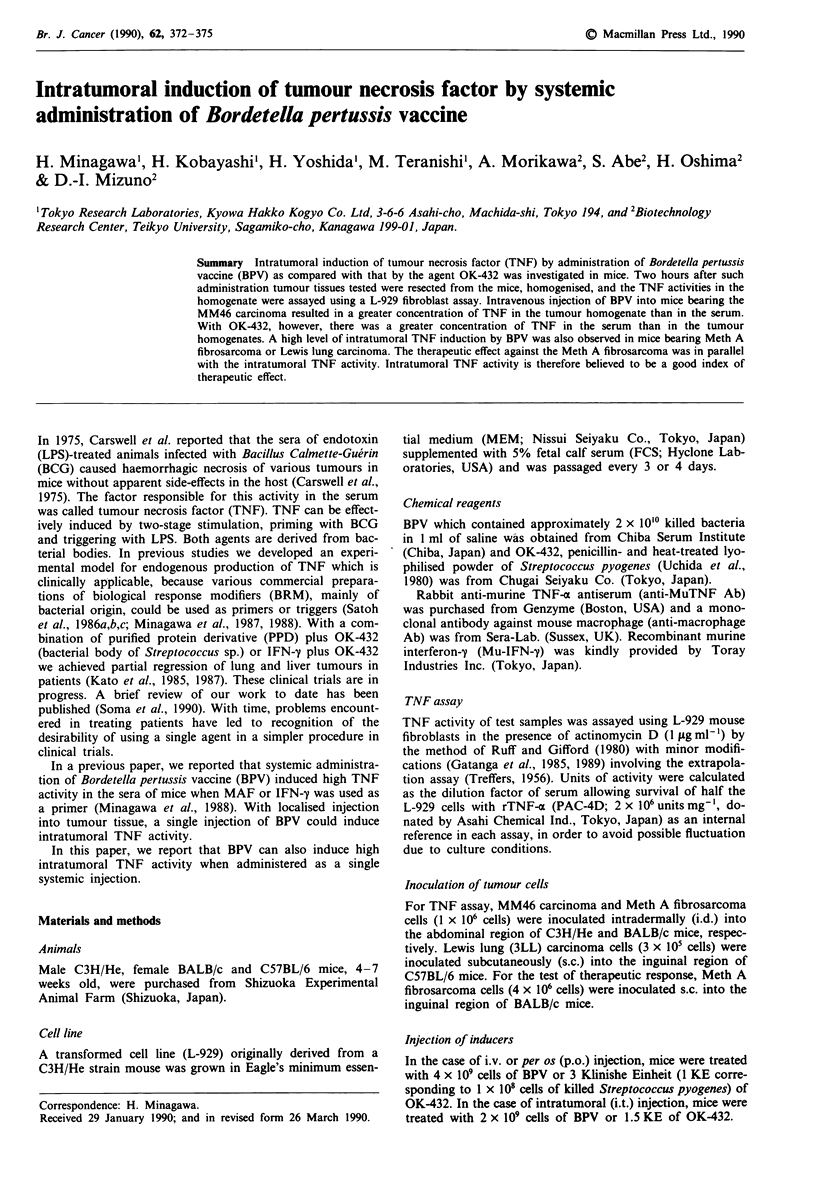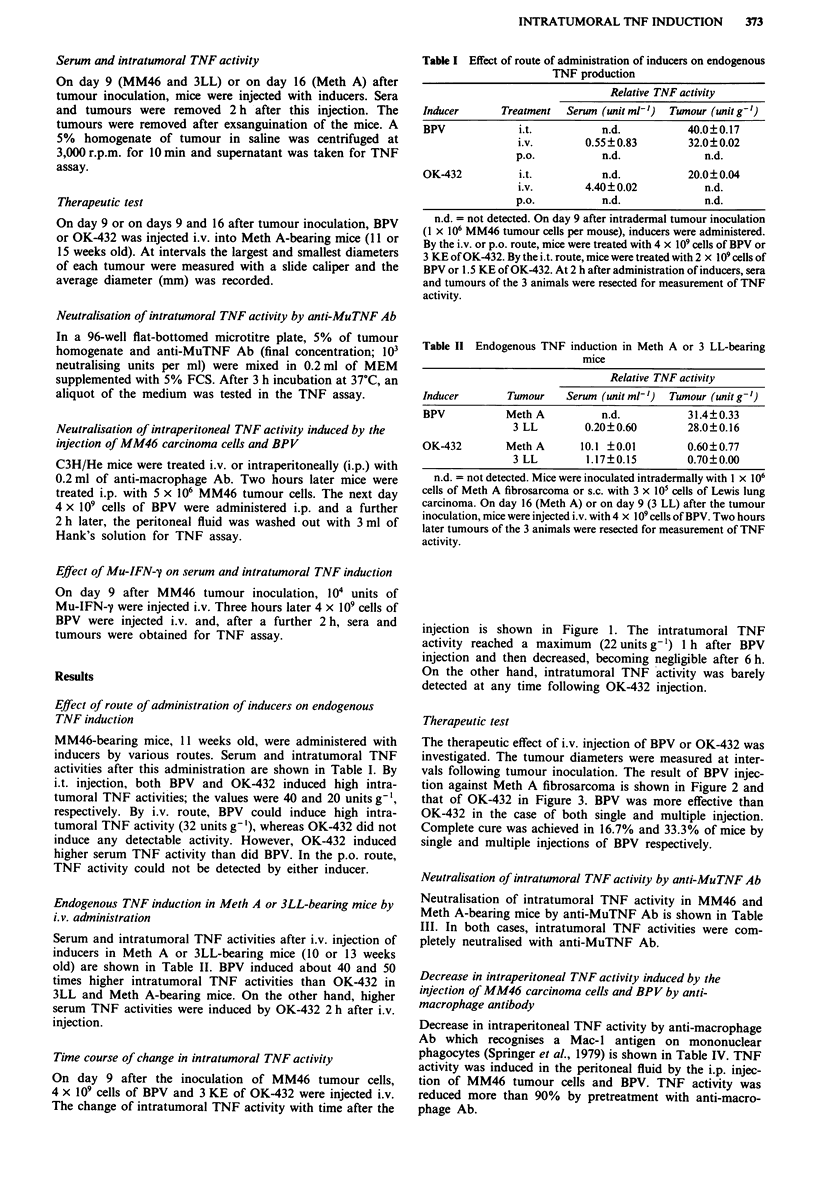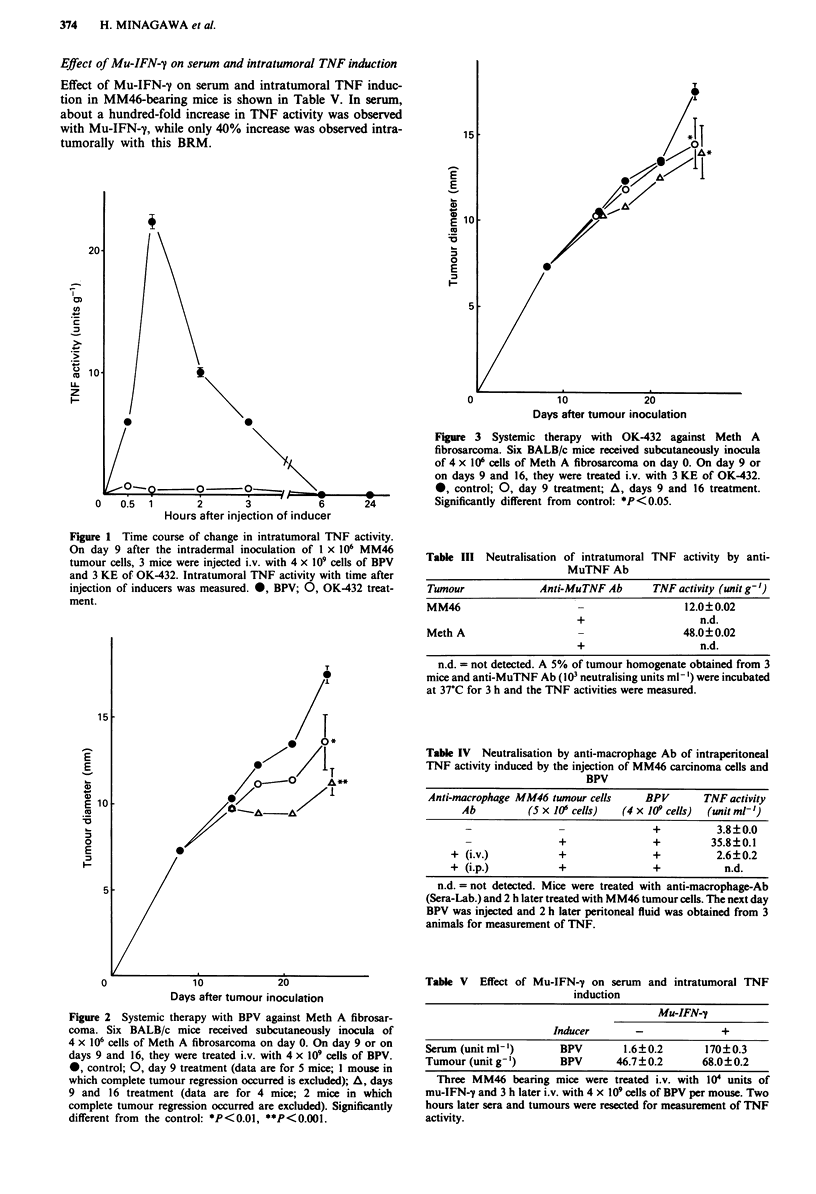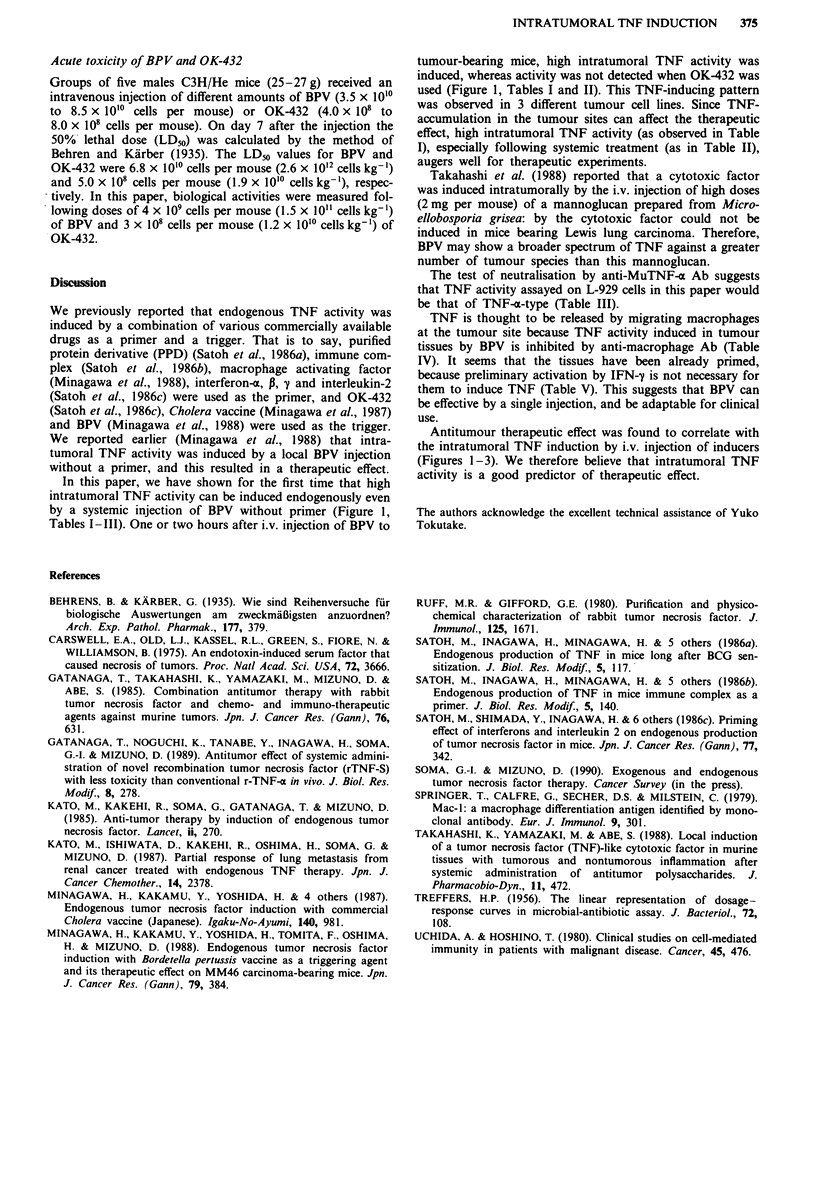# Intratumoral induction of tumour necrosis factor by systemic administration of Bordetella pertussis vaccine.

**DOI:** 10.1038/bjc.1990.300

**Published:** 1990-09

**Authors:** H. Minagawa, H. Kobayashi, H. Yoshida, M. Teranishi, A. Morikawa, S. Abe, H. Oshima, D. I. Mizuno

**Affiliations:** Tokyo Research Laboratories, Kyowa Hakko Kogyo Co. Ltd., Japan.

## Abstract

Intratumoral induction of tumour necrosis factor (TNF) by administration of Bordetella pertussis vaccine (BPV) as compared with that by the agent OK-432 was investigated in mice. Two hours after such administration tumour tissues tested were resected from the mice, homogenised, and the TNF activities in the homogenate were assayed using a L-929 fibroblast assay. Intravenous injection of BPV into mice bearing the MM46 carcinoma resulted in a greater concentration of TNF in the tumour homogenate than in the serum. With OK-432, however, there was a greater concentration of TNF in the serum than in the tumour homogenates. A high level of intratumoral TNF induction by BPV was also observed in mice bearing Meth A fibrosarcoma or Lewis lung carcinoma. The therapeutic effect against the Meth A fibrosarcoma was in parallel with the intratumoral TNF activity. Intratumoral TNF activity is therefore believed to be a good index of therapeutic effect.


Br. J. Cancer (1990), 62, 372-375 © Macmillan Press Ltd., 1990

### Intratumoral induction of tumour necrosis factor by systemic administration of *Bordetella pertussis* vaccine

H. Minagawa[1], H. Kobayashi[1], H. Yoshida[1], M. Teranishi[1], A. Morikawa[2], S. Abe[2], H. Oshima[2] & D.-I. Mizuno[2]

[1]*Tokyo Research Laboratories, Kyowa Hakko Kogyo Co. Ltd, 3-6-6 Asahi-cho, Machida-shi, Tokyo 194, and* [2]*Biotechnology Research Center, Teikyo University, Sagamiko-cho, Kanagawa 199-01, Japan.*

**Summary** Intratumoral induction of tumour necrosis factor (TNF) by administration of *Bordetella pertussis* vaccine (BPV) as compared with that by the agent OK-432 was investigated in mice. Two hours after such administration tumour tissues tested were resected from the mice, homogenised, and the TNF activities in the homogenate were assayed using a L-929 fibroblast assay. Intravenous injection of BPV into mice bearing the MM46 carcinoma resulted in a greater concentration of TNF in the tumour homogenate than in the serum. With OK-432, however, there was a greater concentration of TNF in the serum than in the tumour homogenates. A high level of intratumoral TNF induction by BPV was also observed in mice bearing Meth A fibrosarcoma or Lewis lung carcinoma. The therapeutic effect against the Meth A fibrosarcoma was in parallel with the intratumoral TNF activity. Intratumoral TNF activity is therefore believed to be a good index of therapeutic effect.

In 1975, Carswell *et al.* reported that the sera of endotoxin (LPS)-treated animals infected with *Bacillus Calmette-Guérin* (BCG) caused haemorrhagic necrosis of various tumours in mice without apparent side-effects in the host (Carswell *et al.*, 1975). The factor responsible for this activity in the serum was called tumour necrosis factor (TNF). TNF can be effectively induced by two-stage stimulation, priming with BCG and triggering with LPS. Both agents are derived from bacterial bodies. In previous studies we developed an experimental model for endogenous production of TNF which is clinically applicable, because various commercial preparations of biological response modifiers (BRM), mainly of bacterial origin, could be used as primers or triggers (Satoh *et al.*, 1986*a,b,c*; Minagawa *et al.*, 1987, 1988). With a combination of purified protein derivative (PPD) plus OK-432 (bacterial body of *Streptococcus* sp.) or IFN-γ plus OK-432 we achieved partial regression of lung and liver tumours in patients (Kato *et al.*, 1985, 1987). These clinical trials are in progress. A brief review of our work to date has been published (Soma *et al.*, 1990). With time, problems encountered in treating patients have led to recognition of the desirability of using a single agent in a simpler procedure in clinical trials.

In a previous paper, we reported that systemic administration of *Bordetella pertussis* vaccine (BPV) induced high TNF activity in the sera of mice when MAF or IFN-γ was used as a primer (Minagawa *et al.*, 1988). With localised injection into tumour tissue, a single injection of BPV could induce intratumoral TNF activity.

In this paper, we report that BPV can also induce high intratumoral TNF activity when administered as a single systemic injection.

### Materials and methods

### *Animals*

Male C3H/He, female BALB/c and C57BL/6 mice, 4-7 weeks old, were purchased from Shizuoka Experimental Animal Farm (Shizuoka, Japan).

### *Cell line*

A transformed cell line (L-929) originally derived from a C3H/He strain mouse was grown in Eagle's minimum essential medium (MEM; Nissui Seiyaku Co., Tokyo, Japan) supplemented with 5% fetal calf serum (FCS; Hyclone Laboratories, USA) and was passaged every 3 or 4 days.

### *Chemical reagents*

BPV which contained approximately $2 \times 10^{10}$ killed bacteria in 1 ml of saline was obtained from Chiba Serum Institute (Chiba, Japan) and OK-432, penicillin- and heat-treated lyophilised powder of *Streptococcus pyogenes* (Uchida *et al.*, 1980) was from Chugai Seiyaku Co. (Tokyo, Japan).

Rabbit anti-murine TNF-α antiserum (anti-MuTNF Ab) was purchased from Genzyme (Boston, USA) and a monoclonal antibody against mouse macrophage (anti-macrophage Ab) was from Sera-Lab. (Sussex, UK). Recombinant murine interferon-γ (Mu-IFN-γ) was kindly provided by Toray Industries Inc. (Tokyo, Japan).

### *TNF assay*

TNF activity of test samples was assayed using L-929 mouse fibroblasts in the presence of actinomycin D ($1\ \mu g\ ml^{-1}$) by the method of Ruff and Gifford (1980) with minor modifications (Gatanga *et al.*, 1985, 1989) involving the extrapolation assay (Treffers, 1956). Units of activity were calculated as the dilution factor of serum allowing survival of half the L-929 cells with rTNF-α (PAC-4D; $2 \times 10^6$ units $mg^{-1}$, donated by Asahi Chemical Ind., Tokyo, Japan) as an internal reference in each assay, in order to avoid possible fluctuation due to culture conditions.

### *Inoculation of tumour cells*

For TNF assay, MM46 carcinoma and Meth A fibrosarcoma cells ($1 \times 10^6$ cells) were inoculated intradermally (i.d.) into the abdominal region of C3H/He and BALB/c mice, respectively. Lewis lung (3LL) carcinoma cells ($3 \times 10^5$ cells) were inoculated subcutaneously (s.c.) into the inguinal region of C57BL/6 mice. For the test of therapeutic response, Meth A fibrosarcoma cells ($4 \times 10^6$ cells) were inoculated s.c. into the inguinal region of BALB/c mice.

### *Injection of inducers*

In the case of i.v. or *per os* (p.o.) injection, mice were treated with $4 \times 10^9$ cells of BPV or 3 Klinishe Einheit (1 KE corresponding to $1 \times 10^8$ cells of killed *Streptococcus pyogenes*) of OK-432. In the case of intratumoral (i.t.) injection, mice were treated with $2 \times 10^9$ cells of BPV or 1.5 KE of OK-432.

Correspondence: H. Minagawa.
Received 29 January 1990; and in revised form 26 March 1990.

*Serum and intratumoral TNF activity*

On day 9 (MM46 and 3LL) or on day 16 (Meth A) after tumour inoculation, mice were injected with inducers. Sera and tumours were removed 2 h after this injection. The tumours were removed after exsanguination of the mice. A 5% homogenate of tumour in saline was centrifuged at 3,000 r.p.m. for 10 min and supernatant was taken for TNF assay.

*Therapeutic test*

On day 9 or on days 9 and 16 after tumour inoculation, BPV or OK-432 was injected i.v. into Meth A-bearing mice (11 or 15 weeks old). At intervals the largest and smallest diameters of each tumour were measured with a slide caliper and the average diameter (mm) was recorded.

*Neutralisation of intratumoral TNF activity by anti-MuTNF Ab*

In a 96-well flat-bottomed microtitre plate, 5% of tumour homogenate and anti-MuTNF Ab (final concentration; $10^3$ neutralising units per ml) were mixed in 0.2 ml of MEM supplemented with 5% FCS. After 3 h incubation at 37°C, an aliquot of the medium was tested in the TNF assay.

*Neutralisation of intraperitoneal TNF activity induced by the injection of MM46 carcinoma cells and BPV*

C3H/He mice were treated i.v. or intraperitoneally (i.p.) with 0.2 ml of anti-macrophage Ab. Two hours later mice were treated i.p. with $5 \times 10^6$ MM46 tumour cells. The next day $4 \times 10^9$ cells of BPV were administered i.p. and a further 2 h later, the peritoneal fluid was washed out with 3 ml of Hank's solution for TNF assay.

*Effect of Mu-IFN-γ on serum and intratumoral TNF induction*

On day 9 after MM46 tumour inoculation, $10^4$ units of Mu-IFN-γ were injected i.v. Three hours later $4 \times 10^9$ cells of BPV were injected i.v. and, after a further 2 h, sera and tumours were obtained for TNF assay.

### Results

*Effect of route of administration of inducers on endogenous TNF induction*

MM46-bearing mice, 11 weeks old, were administered with inducers by various routes. Serum and intratumoral TNF activities after this administration are shown in Table I. By i.t. injection, both BPV and OK-432 induced high intratumoral TNF activities; the values were 40 and 20 units $g^{-1}$, respectively. By i.v. route, BPV could induce high intratumoral TNF activity (32 units $g^{-1}$), whereas OK-432 did not induce any detectable activity. However, OK-432 induced higher serum TNF activity than did BPV. In the p.o. route, TNF activity could not be detected by either inducer.

*Endogenous TNF induction in Meth A or 3LL-bearing mice by i.v. administration*

Serum and intratumoral TNF activities after i.v. injection of inducers in Meth A or 3LL-bearing mice (10 or 13 weeks old) are shown in Table II. BPV induced about 40 and 50 times higher intratumoral TNF activities than OK-432 in 3LL and Meth A-bearing mice. On the other hand, higher serum TNF activities were induced by OK-432 2 h after i.v. injection.

*Time course of change in intratumoral TNF activity*

On day 9 after the inoculation of MM46 tumour cells, $4 \times 10^9$ cells of BPV and 3 KE of OK-432 were injected i.v. The change of intratumoral TNF activity with time after the injection is shown in Figure 1. The intratumoral TNF activity reached a maximum (22 units $g^{-1}$) 1 h after BPV injection and then decreased, becoming negligible after 6 h. On the other hand, intratumoral TNF activity was barely detected at any time following OK-432 injection.

**Table I** Effect of route of administration of inducers on endogenous TNF production

| | | Relative TNF activity | |
|---|---|---|---|
| *Inducer* | *Treatment* | *Serum (unit ml⁻¹)* | *Tumour (unit g⁻¹)* |
| BPV | i.t. | n.d. | 40.0±0.17 |
| | i.v. | 0.55±0.83 | 32.0±0.02 |
| | p.o. | n.d. | n.d. |
| OK-432 | i.t. | n.d. | 20.0±0.04 |
| | i.v. | 4.40±0.02 | n.d. |
| | p.o. | n.d. | n.d. |

n.d. = not detected. On day 9 after intradermal tumour inoculation ($1 \times 10^6$ MM46 tumour cells per mouse), inducers were administered. By the i.v. or p.o. route, mice were treated with $4 \times 10^9$ cells of BPV or 3 KE of OK-432. By the i.t. route, mice were treated with $2 \times 10^9$ cells of BPV or 1.5 KE of OK-432. At 2 h after administration of inducers, sera and tumours of the 3 animals were resected for measurement of TNF activity.

**Table II** Endogenous TNF induction in Meth A or 3 LL-bearing mice

| | | Relative TNF activity | |
|---|---|---|---|
| *Inducer* | *Tumour* | *Serum (unit ml⁻¹)* | *Tumour (unit g⁻¹)* |
| BPV | Meth A | n.d. | 31.4±0.33 |
| | 3 LL | 0.20±0.60 | 28.0±0.16 |
| OK-432 | Meth A | 10.1 ±0.01 | 0.60±0.77 |
| | 3 LL | 1.17±0.15 | 0.70±0.00 |

n.d. = not detected. Mice were inoculated intradermally with $1 \times 10^6$ cells of Meth A fibrosarcoma or s.c. with $3 \times 10^5$ cells of Lewis lung carcinoma. On day 16 (Meth A) or on day 9 (3 LL) after the tumour inoculation, mice were injected i.v. with $4 \times 10^9$ cells of BPV. Two hours later tumours of the 3 animals were resected for measurement of TNF activity.

*Therapeutic test*

The therapeutic effect of i.v. injection of BPV or OK-432 was investigated. The tumour diameters were measured at intervals following tumour inoculation. The result of BPV injection against Meth A fibrosarcoma is shown in Figure 2 and that of OK-432 in Figure 3. BPV was more effective than OK-432 in the case of both single and multiple injection. Complete cure was achieved in 16.7% and 33.3% of mice by single and multiple injections of BPV respectively.

*Neutralisation of intratumoral TNF activity by anti-MuTNF Ab*

Neutralisation of intratumoral TNF activity in MM46 and Meth A-bearing mice by anti-MuTNF Ab is shown in Table III. In both cases, intratumoral TNF activities were completely neutralised with anti-MuTNF Ab.

*Decrease in intraperitoneal TNF activity induced by the injection of MM46 carcinoma cells and BPV by anti-macrophage antibody*

Decrease in intraperitoneal TNF activity by anti-macrophage Ab which recognises a Mac-1 antigen on mononuclear phagocytes (Springer *et al.*, 1979) is shown in Table IV. TNF activity was induced in the peritoneal fluid by the i.p. injection of MM46 tumour cells and BPV. TNF activity was reduced more than 90% by pretreatment with anti-macrophage Ab.

*Effect of Mu-IFN-γ on serum and intratumoral TNF induction*

Effect of Mu-IFN-γ on serum and intratumoral TNF induction in MM46-bearing mice is shown in Table V. In serum, about a hundred-fold increase in TNF activity was observed with Mu-IFN-γ, while only 40% increase was observed intratumorally with this BRM.

**Figure 1** Time course of change in intratumoral TNF activity. On day 9 after the intradermal inoculation of 1 × $10^6$ MM46 tumour cells, 3 mice were injected i.v. with 4 × $10^9$ cells of BPV and 3 KE of OK-432. Intratumoral TNF activity with time after injection of inducers was measured. ●, BPV; ○, OK-432 treatment.

**Figure 2** Systemic therapy with BPV against Meth A fibrosarcoma. Six BALB/c mice received subcutaneously inocula of 4 × $10^6$ cells of Meth A fibrosarcoma on day 0. On day 9 or on days 9 and 16, they were treated i.v. with 4 × $10^9$ cells of BPV. ●, control; ○, day 9 treatment (data are for 5 mice; 1 mouse in which complete tumour regression occurred is excluded); Δ, days 9 and 16 treatment (data are for 4 mice; 2 mice in which complete tumour regression occurred are excluded). Significantly different from the control: $^*P<0.01$, $^{**}P<0.001$.

**Figure 3** Systemic therapy with OK-432 against Meth A fibrosarcoma. Six BALB/c mice received subcutaneously inocula of 4 × $10^6$ cells of Meth A fibrosarcoma on day 0. On day 9 or on days 9 and 16, they were treated i.v. with 3 KE of OK-432. ●, control; ○, day 9 treatment; Δ, days 9 and 16 treatment. Significantly different from control: $^*P<0.05$.

**Table III** Neutralisation of intratumoral TNF activity by anti-MuTNF Ab

| *Tumour* | *Anti-MuTNF Ab* | *TNF activity (unit $g^{-1}$)* |
|---|---|---|
| MM46 | − | 12.0±0.02 |
| | + | n.d. |
| Meth A | − | 48.0±0.02 |
| | + | n.d. |

n.d. = not detected. A 5% of tumour homogenate obtained from 3 mice and anti-MuTNF Ab ($10^3$ neutralising units $ml^{-1}$) were incubated at 37°C for 3 h and the TNF activities were measured.

**Table IV** Neutralisation by anti-macrophage Ab of intraperitoneal TNF activity induced by the injection of MM46 carcinoma cells and BPV

| *Anti-macrophage Ab* | *MM46 tumour cells (5 × $10^6$ cells)* | *BPV (4 × $10^9$ cells)* | *TNF activity (unit $ml^{-1}$)* |
|---|---|---|---|
| − | − | + | 3.8±0.0 |
| − | + | + | 35.8±0.1 |
| + (i.v.) | + | + | 2.6±0.2 |
| + (i.p.) | + | + | n.d. |

n.d. = not detected. Mice were treated with anti-macrophage-Ab (Sera-Lab.) and 2 h later treated with MM46 tumour cells. The next day BPV was injected and 2 h later peritoneal fluid was obtained from 3 animals for measurement of TNF.

**Table V** Effect of Mu-IFN-γ on serum and intratumoral TNF induction

| | | *Mu-IFN-γ* | |
|---|---|---|---|
| | *Inducer* | − | + |
| Serum (unit $ml^{-1}$) | BPV | 1.6±0.2 | 170±0.3 |
| Tumour (unit $g^{-1}$) | BPV | 46.7±0.2 | 68.0±0.2 |

Three MM46 bearing mice were treated i.v. with $10^4$ units of mu-IFN-γ and 3 h later i.v. with 4 × $10^9$ cells of BPV per mouse. Two hours later sera and tumours were resected for measurement of TNF activity.

*Acute toxicity of BPV and OK-432*

Groups of five males C3H/He mice (25–27 g) received an intravenous injection of different amounts of BPV ($3.5 \times 10^{10}$ to $8.5 \times 10^{10}$ cells per mouse) or OK-432 ($4.0 \times 10^8$ to $8.0 \times 10^8$ cells per mouse). On day 7 after the injection the 50% lethal dose ($LD_{50}$) was calculated by the method of Behren and Kärber (1935). The $LD_{50}$ values for BPV and OK-432 were $6.8 \times 10^{10}$ cells per mouse ($2.6 \times 10^{12}$ cells $kg^{-1}$) and $5.0 \times 10^8$ cells per mouse ($1.9 \times 10^{10}$ cells $kg^{-1}$), respectively. In this paper, biological activities were measured following doses of $4 \times 10^9$ cells per mouse ($1.5 \times 10^{11}$ cells $kg^{-1}$) of BPV and $3 \times 10^8$ cells per mouse ($1.2 \times 10^{10}$ cells $kg^{-1}$) of OK-432.

### Discussion

We previously reported that endogenous TNF activity was induced by a combination of various commercially available drugs as a primer and a trigger. That is to say, purified protein derivative (PPD) (Satoh *et al.*, 1986*a*), immune complex (Satoh *et al.*, 1986*b*), macrophage activating factor (Minagawa *et al.*, 1988), interferon-α, β, γ and interleukin-2 (Satoh *et al.*, 1986*c*) were used as the primer, and OK-432 (Satoh *et al.*, 1986*c*), *Cholera* vaccine (Minagawa *et al.*, 1987) and BPV (Minagawa *et al.*, 1988) were used as the trigger. We reported earlier (Minagawa *et al.*, 1988) that intratumoral TNF activity was induced by a local BPV injection without a primer, and this resulted in a therapeutic effect.

In this paper, we have shown for the first time that high intratumoral TNF activity can be induced endogenously even by a systemic injection of BPV without primer (Figure 1, Tables I–III). One or two hours after i.v. injection of BPV to tumour-bearing mice, high intratumoral TNF activity was induced, whereas activity was not detected when OK-432 was used (Figure 1, Tables I and II). This TNF-inducing pattern was observed in 3 different tumour cell lines. Since TNF-accumulation in the tumour sites can affect the therapeutic effect, high intratumoral TNF activity (as observed in Table I), especially following systemic treatment (as in Table II), augers well for therapeutic experiments.

Takahashi *et al.* (1988) reported that a cytotoxic factor was induced intratumorally by the i.v. injection of high doses (2 mg per mouse) of a mannoglucan prepared from *Microellobosporia grisea*: by the cytotoxic factor could not be induced in mice bearing Lewis lung carcinoma. Therefore, BPV may show a broader spectrum of TNF against a greater number of tumour species than this mannoglucan.

The test of neutralisation by anti-MuTNF-α Ab suggests that TNF activity assayed on L-929 cells in this paper would be that of TNF-α-type (Table III).

TNF is thought to be released by migrating macrophages at the tumour site because TNF activity induced in tumour tissues by BPV is inhibited by anti-macrophage Ab (Table IV). It seems that the tissues have been already primed, because preliminary activation by IFN-γ is not necessary for them to induce TNF (Table V). This suggests that BPV can be effective by a single injection, and be adaptable for clinical use.

Antitumour therapeutic effect was found to correlate with the intratumoral TNF induction by i.v. injection of inducers (Figures 1–3). We therefore believe that intratumoral TNF activity is a good predictor of therapeutic effect.

The authors acknowledge the excellent technical assistance of Yuko Tokutake.

### References

BEHRENS, B. & KÄRBER, G. (1935). Wie sind Reihenversuche für biologische Auswertungen am zweckmäßigsten anzuordnen? *Arch. Exp. Pathol. Pharmak.*, **177**, 379.

CARSWELL, E.A., OLD, L.J., KASSEL, R.L., GREEN, S., FIORE, N. & WILLIAMSON, B. (1975). An endotoxin-induced serum factor that caused necrosis of tumors. *Proc. Natl Acad. Sci. USA*, **72**, 3666.

GATANAGA, T., TAKAHASHI, K., YAMAZAKI, M., MIZUNO, D. & ABE, S. (1985). Combination antitumor therapy with rabbit tumor necrosis factor and chemo- and immuno-therapeutic agents against murine tumors. *Jpn. J. Cancer Res. (Gann)*, **76**, 631.

GATANAGA, T., NOGUCHI, K., TANABE, Y., INAGAWA, H., SOMA, G.-I. & MIZUNO, D. (1989). Antitumor effect of systemic administration of novel recombination tumor necrosis factor (rTNF-S) with less toxicity than conventional r-TNF-α *in vivo*. *J. Biol. Res. Modif.*, **8**, 278.

KATO, M., KAKEHI, R., SOMA, G., GATANAGA, T. & MIZUNO, D. (1985). Anti-tumor therapy by induction of endogenous tumor necrosis factor. *Lancet*, **ii**, 270.

KATO, M., ISHIWATA, D., KAKEHI, R., OSHIMA, H., SOMA, G. & MIZUNO, D. (1987). Partial response of lung metastasis from renal cancer treated with endogenous TNF therapy. *Jpn. J. Cancer Chemother.*, **14**, 2378.

MINAGAWA, H., KAKAMU, Y., YOSHIDA, H. & 4 others (1987). Endogenous tumor necrosis factor induction with commercial *Cholera* vaccine (Japanese). *Igaku-No-Ayumi*, **140**, 981.

MINAGAWA, H., KAKAMU, Y., YOSHIDA, H., TOMITA, F., OSHIMA, H. & MIZUNO, D. (1988). Endogenous tumor necrosis factor induction with *Bordetella pertussis* vaccine as a triggering agent and its therapeutic effect on MM46 carcinoma-bearing mice. *Jpn. J. Cancer Res. (Gann)*, **79**, 384.

RUFF, M.R. & GIFFORD, G.E. (1980). Purification and physicochemical characterization of rabbit tumor necrosis factor. *J. Immunol.*, **125**, 1671.

SATOH, M., INAGAWA, H., MINAGAWA, H. & 5 others (1986*a*). Endogenous production of TNF in mice long after BCG sensitization. *J. Biol. Res. Modif.*, **5**, 117.

SATOH, M., INAGAWA, H., MINAGAWA, H. & 5 others (1986*b*). Endogenous production of TNF in mice immune complex as a primer. *J. Biol. Res. Modif.*, **5**, 140.

SATOH, M., SHIMADA, Y., INAGAWA, H. & 6 others (1986*c*). Priming effect of interferons and interleukin 2 on endogenous production of tumor necrosis factor in mice. *Jpn. J. Cancer Res. (Gann)*, **77**, 342.

SOMA, G.-I. & MIZUNO, D. (1990). Exogenous and endogenous tumor necrosis factor therapy. *Cancer Survey* (in the press).

SPRINGER, T., CALFRE, G., SECHER, D.S. & MILSTEIN, C. (1979). Mac-1: a macrophage differentiation antigen identified by monoclonal antibody. *Eur. J. Immunol.* **9**, 301.

TAKAHASHI, K., YAMAZAKI, M. & ABE, S. (1988). Local induction of a tumor necrosis factor (TNF)-like cytotoxic factor in murine tissues with tumorous and nontumorous inflammation after systemic administration of antitumor polysaccharides. *J. Pharmacobio-Dyn.*, **11**, 472.

TREFFERS, H.P. (1956). The linear representation of dosage–response curves in microbial-antibiotic assay. *J. Bacteriol.*, **72**, 108.

UCHIDA, A. & HOSHINO, T. (1980). Clinical studies on cell-mediated immunity in patients with malignant disease. *Cancer*, **45**, 476.